# Complete genome sequence of *Rhodococcus erythropolis* strain ICBD2, isolated from hydrocarbon-polluted coastal sediments in Central Chile

**DOI:** 10.1128/mra.00314-26

**Published:** 2026-05-29

**Authors:** Ximena Báez-Matus, Roberto E. Durán, Roberto Orellana, Andrés Cumsille, Ester G. Rivera, Teresa Esparza-Correa, Francisco Salvà-Serra, Daniel Jaén-Luchoro, Edward R. B. Moore, Michael Seeger

**Affiliations:** 1 Molecular Microbiology and Environmental Biotechnology Laboratory, Department of Chemistry and Center of Biotechnology Daniel Alkalay Lowitt, Universidad Técnica Federico Santa María, Valparaíso, Chile; 2 Millennium Nucleus Bioproducts, Genomics and Environmental Microbiology (BioGEM), Valparaíso, Chile; 3 Laboratorio de Biología Celular y Ecofisiología Microbiana, Facultad de Ciencias Naturales y Exactas, Universidad de Playa Ancha, Valparaíso, Chile; 4 HUB Ambiental UPLA, Universidad de Playa Ancha, Valparaíso, Chile; 5 Department of Infectious Diseases, Institute of Biomedicine, University of Gothenburg, Sahlgrenska Academy, Gothenburg, Sweden; 6 Centre for Antibiotic Resistance Research (CARe), University of Gothenburg, Gothenburg, Sweden; 7 Culture Collection University of Gothenburg (CCUG), Department of Clinical Microbiology, Sahlgrenska University Hospital, Region Västra Götaland and Sahlgrenska Academy, University of Gothenburg 378992, Gothenburg, Sweden; 8 Unit of Medical Technology and Biological Function, Department of Medical Technology and Diagnostics, RISE Research Institutes of Sweden, Mölndal, Sweden; University of Manitoba, Winnipeg, Manitoba, Canada

**Keywords:** *Rhodococcus erythropolis*, genome, hydrocarbon, Central Chile

## Abstract

*Rhodococcus erythropolis* strain ICBD2 (=CCUG 74281) is a hydrocarbon-degrading bacterium isolated from chronically hydrocarbon-polluted saline coastal sediments from the Valparaíso Region, Central Chile. The complete genome sequence of *R. erythropolis* ICBD2 (62.3% GC content) comprises a 6.35 Mb circular chromosome and a 387 kb linear plasmid, with 6,196 coding sequences.

## ANNOUNCEMENT

*Rhodococcus* genus (*Actinomycetota* phylum) comprises aerobic, non-motile, metabolically versatile Gram-positive bacteria that degrade persistent organic pollutants and synthesize antimicrobial molecules (1–4). Environmental bacteria possess diverse adaptation mechanisms to oxidative and saline stress (5–7). This study presents the complete genome sequence of the hydrocarbon-degrading bacterium *Rhodococcus erythropolis* ICBD2 (=CCUG 74281), isolated from a chronically hydrocarbon-polluted saline coastal sediment at the Las Salinas site (33.0011° S, 71.5469° W) in Viña del Mar (Valparaíso Region, Central Chile). Strain ICBD2 was isolated in 2016 after two successive enrichments of a sediment sample (1 g, 4 m depth) in Bushnell–Haas (BH) mineral medium (20 mL) using diesel (1% vol/vol) and acetate (10 mM) as sole carbon/energy sources without agitation. Thereafter, the enrichment culture without prior dilution (100 µL) was plated onto BH agar containing acetate (10 mM) as sole carbon/energy sources; a single colony was isolated and characterized. Strain ICBD2 grew in BH or low-phosphate Tris-buffered mineral salt media on *n*-dodecane, *n*-tetradecane, *n*-hexadecane, *n*-eicosane, and diesel as sole carbon/energy sources (30°C).

Genomic DNA was extracted from a single colony grown on tryptic soy agar using a modified Marmur procedure (8). An Illumina standard genomic paired-end library, prepared from DNA using an optimized protocol (Eurofins Genomics, Konstanz, Germany) with standard Illumina adapters, was Illumina-sequenced (NovaSeq-6000-platform/flow-cell S2), achieving 22,030,486 paired-end reads/151 bp average length (3,326 Mb). Quality was assessed using FastQC (v0.11.9) (9). DNA was complementary sequenced using the Oxford Nanopore Technologies MinION Mk101B sequencer. The library was prepared with DNA without pre-treatment and Rapid-Barcoding-Kit (vR9) (SQK-RBK004) and Nanopore-sequenced (48 h) using a flow cell FLO-MIN106 (vR9.4). Base calling was done (Guppy, v4.0.14), obtaining 157,563 long reads/10,937 bp average length. The hybrid assembly using SPAdes (v3.13.1) with read error correction (10) yielded a single circular chromosome (6,351,314 bp/62.3% GC) and a linear plasmid (387,366 bp/61.1% GC), containing inverted repeats at the ends. The overlaps were identified by BLASTN analysis of the ends against the genome sequence. The overlap of one of the chromosome ends was trimmed manually using UGENE (v36). The *dnaA* gene located by TBLASTN analysis of the Unicycler (v0.4.8) start gene database was chosen as the start gene to re-orient the chromosome (UGENE v36). Genome completeness (CheckM2, v1.0.2) was assessed (11). Genes were annotated using the NCBI Prokaryotic Genome Annotation Pipeline (v6.10) (12). All software was utilized with default parameters unless otherwise noted. The ICBD2 genome (100% completeness) contains 6,196 coding sequences, 54 tRNA genes, and 15 rRNA genes.

Comparative whole-genome sequence analyses with the Type Strain Genome Server platform (13) and Pyani-plus (v.1.0.0) (14) identified strain ICBD2 as *Rhodococcus erythropolis*; 16S rRNA gene identity (100%), digital DNA–DNA hybridization (*d*4 = 89.3%), and average nucleotide identity (ANIb = 98.5%) with *Rhodococcus erythropolis* NBRC 15567^T^.

BLAST gene function analyses using the UniProt-KB/Swiss-Prot database (>30% identity) identified genes associated with alkane degradation and oxidative stress response, including five *alkB* genes encoding alkane monooxygenase (≥69% identity with *Rhodococcus* spp. *alkB* genes, ACV96D_03880/ACV96D_10690/ACV96D_26645/ACV96D_11635/ACV96D_31060), *katG* gene encoding catalase-peroxidase (ACV96D_05890), *katA* (ACV96D_02200), and *katE* (ACV96D_28135) genes encoding catalases, and *sod* genes encoding superoxide dismutase ([Mn]SodA/ACV96D_00775, [Fe]SodB/ACV96D_23855, [Cu-Zn]SodC/ACV96D_07375). The *Rhodococcus erythropolis* ICBD2 genetic repertoire highlights its potential for hydrocarbon biodegradation.

## Data Availability

The complete genome sequence of *Rhodococcus erythropolis* ICBD2 has been deposited in DDBJ/ENA/GenBank under accession number JBSDZJ000000000. The genome sequence described in this paper is the first version, BioProject PRJNA1309126. The raw sequencing reads are deposited and publicly available at the Sequence Read Archive, under accession numbers SRR35078506 (MiniON) and SRR35078507 (Illumina).
